# Targeting Imperfect Vaccines against Drug-Resistance Determinants: A Strategy for Countering the Rise of Drug Resistance

**DOI:** 10.1371/journal.pone.0068940

**Published:** 2013-07-25

**Authors:** Regina Joice, Marc Lipsitch

**Affiliations:** 1 Department of Immunology and Infectious Diseases, Harvard School of Public Health, Boston, Massachusetts, United States of America; 2 Center for Communicable Disease Dynamics and Department of Epidemiology, Harvard School of Public Health, Boston, Massachusetts, United States of America; University of Oxford, Viet Nam

## Abstract

The growing prevalence of antimicrobial resistance in major pathogens is outpacing discovery of new antimicrobial classes. Vaccines mitigate the effect of antimicrobial resistance by reducing the need for treatment, but vaccines for many drug-resistant pathogens remain undiscovered or have limited efficacy, in part because some vaccines selectively favor pathogen strains that escape vaccine-induced immunity. A strain with even a modest advantage in vaccinated hosts can have high fitness in a population with high vaccine coverage, which can offset a strong selection pressure such as antimicrobial use that occurs in a small fraction of hosts. We propose a strategy to target vaccines against drug-resistant pathogens, by using resistance-conferring proteins as antigens in multicomponent vaccines. Resistance determinants may be weakly immunogenic, offering only modest specific protection against resistant strains. Therefore, we assess here how varying the specific efficacy of the vaccine against resistant strains would affect the proportion of drug-resistant vs. –sensitive strains population-wide for three pathogens – *Streptococcus pneumoniae*, *Staphylococcus aureus*, and influenza virus – in which drug resistance is a problem. Notably, if such vaccines confer even slightly higher protection (additional efficacy between 1% and 8%) against resistant variants than sensitive ones, they may be an effective tool in controlling the rise of resistant strains, given current levels of use for many antimicrobial agents. We show that the population-wide impact of such vaccines depends on the additional effect on resistant strains and on the overall effect (against all strains). Resistance-conferring accessory gene products or resistant alleles of essential genes could be valuable as components of vaccines even if their specific protective effect is weak.

## Introduction

Increasing antimicrobial resistance in pathogen populations results from concurrent selective processes [1]: emergence of resistant strains in treated hosts and the differential transmission success of resistant and sensitive strains. The latter effect becomes increasingly important as the prevalence of resistant strains grows [2]. Selection for resistance is often counteracted in untreated hosts by a fitness cost of resistance: reduced viability, infectiousness, replication, or transmissibility of resistant strains relative to sensitive ones [3]. Simple models for the spread of resistance in populations suggest that the prevalence of resistance will increase when selection for resistance by antimicrobial use outweighs the fitness cost, and will decline otherwise [4], [5]. A corollary of this prediction is that, if both antimicrobial use and fitness cost remain constant, then resistance prevalence should either remain near zero (as in the case of penicillin resistance for Group A Streptococcus [6] or, in some countries, fluoroquinolone resistance in *Streptococcus pneumoniae* [7], or increase to approach 100% with time, as has been the case in some pathogens (e.g. penicillin resistance in *Staphylococcus aureus* [8], adamantane resistance in influenza A/H3N2 [9] and oseltamivir resistance in influenza A/H1N1 before 2009 [10]). In other pathogens, these scenarios do not hold: the prevalence of resistance appears to stabilize at intermediate values (e.g. resistance to several antimicrobial classes in *Streptococcus pneumoniae* [11] or *Neisseria gonorrhoeae* [12]); more complex models are required to explain such coexistence [11].

Vaccines are a key tool in the fight against resistant pathogens. Some vaccines (e.g. diphtheria vaccine) can eliminate their targeted disease in a population (via direct and herd-immunity effects) and thereby obviate the need for treatment and concern about resistance. Other vaccines, such as current vaccines against pneumococcal disease and influenza, cannot eliminate transmission because their uptake or efficacy against colonization/infection are too low, and/or because they do not cover all strains of their target pathogen. By reducing disease burden, they nonetheless reduce the need for treatment [13] and may thereby reduce the selective pressure for resistance. Moreover, they reduce the impact of resistance, since fewer cases treated means fewer instances in which treatment can fail due to resistance. Intriguingly, pneumococcal conjugate vaccination had another benefit: the incidence of drug-resistant infection declined disproportionately because the pneumococcal serotypes in the vaccine tended to be more drug-resistant than those excluded from the vaccine [14]. Unfortunately, as resistance has grown in non-vaccine types, this benefit has waned, so that resistance prevalence is returning to pre-vaccine levels [14], [15], although total disease burden has declined.

If this feature of the pneumococcal conjugate vaccine – disproportionate efficacy against drug-resistant strains of a pathogen – could be made a permanent feature of pneumococcal or other vaccines, then these vaccines could be a tool to increase the fitness cost of resistance and possibly tip the balance between antimicrobial selection and fitness cost in favor of the drug-sensitive strains. Pathogen moieties conferring drug resistance have not been popular targets for vaccines, perhaps because many drug resistance determinants are poorly accessible to antibodies. However, some important resistance determinants could be targets of vaccination. Penicillin binding protein 2 (PBP2) of *Neisseria meningitidis* is immunogenic and protective in a mouse model [16]. Whole-virus influenza vaccines induce immune responses to neuraminidase [17], the target of oseltamivir and other neuraminidase inhibitors, which is altered in oseltamivir-resistant strains [10]. Porins or efflux pumps that are altered (or uniquely present) on the surface of resistant strains [18], [19], [20], [21] might be accessible to antibodies or elicit T cell responses. Certain efflux pumps of *Mycobacterium tuberculosis* appear to contain T cell epitopes [22] and vaccines that protect by inducing T cells [23], [24] might be capable of targeting even intracellular resistance-determining moieties, such as antibiotic-modifying or target-modifying enzymes. Given the often-subtle genetic changes encoding resistance in targets such as PBP2 or neuraminidase, immunity induced by vaccination with a resistant allele of these determinants might be only modestly more effective against resistant variants than against sensitive variants. Yet a strain with even a modest advantage in vaccinated hosts can have high fitness in a population with high vaccine coverage, because the advantage will be realized in a high proportion of hosts. In contrast, the selective effect of antimicrobial use is to exert lethal selection against drug-sensitive strains in the subset of infections that are treated, but this selection is felt in relatively few hosts for organisms that are often carried asymptomatically or that cause self-limiting infections.

The concept of using ecological approaches for eliminating drug resistance (i.e. interventions designed to decrease the proportion of drug-resistant strains in favor of drug-sensitive ones) has been previously discussed [25], though this is still an underdeveloped area of research. Traditional interventions to combat drug-resistance involve infection control, which may be disproportionately effective against resistant strains [26], killing drug-resistant pathogens with new antimicrobial therapeutics, and preventing the emergence of drug resistance in patients through the administration of combination therapies, respectively. Ecological approaches to combat drug resistance have been proposed less frequently, including the use of vaccines or bacteriophages that target specific antigens of the most transmissible and/or drug-resistant clones [25].

Mathematical models have been used to study vaccine-induced strain replacement as it relates to drug resistance in two studies (in pneumococcus [27] and recently in *S. aureus* in hospital outbreaks [28]). In the former, the authors model wide-scale childhood immunization with a pneumococcal conjugate vaccine targeting drug-resistant serotypes. The model successfully predicts a transient reduction in drug-resistance population-wide that is *not* sustained long term [27], as has also been observed in epidemiological studies [14], [15]. The explanation for this phenomenon is the increase in the rate of carriage of non-vaccine serotypes among vaccinated individuals (serotype replacement) paired with the increase in drug-resistance among these non-vaccine serotypes, a phenomenon that does appear to be underway in the US [29]. Thus, the authors argue that targeting drug-resistant strains for vaccination will not achieve a sustained reduction in drug-resistance. In a second modeling study, the authors model hospital-based immunization with a vaccine targeting a resistant strain of *S. aureus*. The model predicts the elimination of drug-resistance in a closed hospital setting, but in an open hospital setting with a constant flow of newly admitted patients, drug-resistance is shown to remain constant despite immunization [28]. This observation is explained by the lag time required to induce protective immunity following vaccination, compared with the influx of new non-vaccinated patients.

Previous models do not clearly support a role for vaccinating against drug-resistant strains in achieving sustainable population-wide reductions in drug-resistance. Thus here we model vaccination against resistance-conferring proteins themselves, such that reduction of vaccine-targeted strains remains permanently linked to those strains containing drug-resistance determinants. We model this situation in a wide-scale vaccination scenario in order to test the effectiveness of such vaccination population-wide, and model conditions for three diverse microbes. Specifically, we test the possibility that modest differential effectiveness due to vaccination with resistance determinants could lead to substantial selective pressure at the population level, sufficient to offset realistic levels of selection from antimicrobial use.

## Materials and Methods

For the pnemococcal vaccine model, we employed a susceptible-infectious-susceptible (SIS) model that allows individuals to be colonized with both drug-sensitive and -resistant strains and permits coexistence of both strains. Drug-resistant strains start out as a minority of all strains (consistent with penicillin-resistance prevalence of 24% in the U.S.) [30], in the presence of high levels of antibiotic usage (consistent with that of young children in the U.S.) [31], and in the presence of a vaccine with additional efficacy against drug resistance determinants. We assumed 80% coverage of the vaccine in the segment of the population responsible for most transmission, as would be realized after 4–5 years of infant vaccination if the core group were children under 5 [14], [32].

For the staphylococcal vaccine, we used an SIS model in which individuals can be colonized by either a drug-sensitive or a drug-resistant strain, but not both. We modeled the rate of treatment that clears MSSA (but not MRSA) in the community as 50% of the MSSA-specific antibiotic prescriptions in the U.S [33], to account for the fact that not all antimicrobial treatment clears carriage, and modeled fitness costs within the range previously measured for methicillin-resistant *S. aureus* [34]. The system of differential equations has four equilibrium states (i. sensitive strain reaches 100%, ii. resistant strain reaches 100%, iii. coexistence and iv. elimination of both strains); we identified stability conditions using eigenvalues of the Jacobian matrix (see Supporting Information). We assessed a range of parameter values required to eliminate resistance by plotting equilibrium stability conditions as a function of resistant strain-specific vaccine efficacy (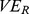) and vaccine coverage (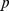) and for multiple different fitness costs within the range previously measured for MRSA [34].

For the influenza vaccine, the model structure and parameter values were taken from Ref. [2] except that 30% of the population (all ages) was assumed immune due to prior exposure at the start of the season, a fraction 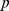 of the population were vaccinated at the start of the season, and the vaccine was 59% effective [35] against infection with the drug-sensitive virus, with an additional efficacy 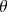 against resistant infection.

The systems of equations for each model are provided in the Supporting Information, and parameter values are provided in Table 1.

**Table 1:** Parameter Values.

Symbol	Description	*S. pneumo* value	*S. aureus* value	Flu value
*β*	Transmission rate/week	**0.4167** (based on 30–50% prevalence [58])	**0.0893** (based on 30% prevalence [41])	Varies by age, see [2]
*τ*	Treatment rate/week	**0.02** (lower end of childhood antibiotic prescribing range [59])	**0.0003, 0.0017, 0.0033** (10, 50, and 100% respectively of MSSA-active antibiotic prescriptions per person per week in the U.S., weighted by ages in 2010 census data [33])	**40**% of infections are treated
*u*	Clearance rate/week	**0.25** (duration of 30 days [60], [61])	**0.01, 0.02, 0.04** (duration of 175–700 days [56], [57])	**2.1** (duration of 3.3 days)
*p*	Proportion of population that is vaccinated	**0.8** (well below U.S. childhood vaccination rates [62])	Range: **0–1**	**0.4**
*VE*	Vaccine efficacy (overall)	Range: **0–1**	n/a	**59**% (effects on infectiousness neglected)
*θ* or *VE_R_*	Additional vaccine efficacy against resistant strain (*θ*, *S. pneumo*, Flu) or total vaccine efficacy against resistant strain only (VE_R_, *S. aureus*)	Range: **0–1**	Range: **0–1**	Range: **0–20**%
*ψ*	Fitness cost	Range: **0, 0.08** (estimated based on *in vitro* data [36])	**0.02, 0.04, 0.08** (range of fitness costs in field strains [34])	**0**

## Results

**Figure 1:**
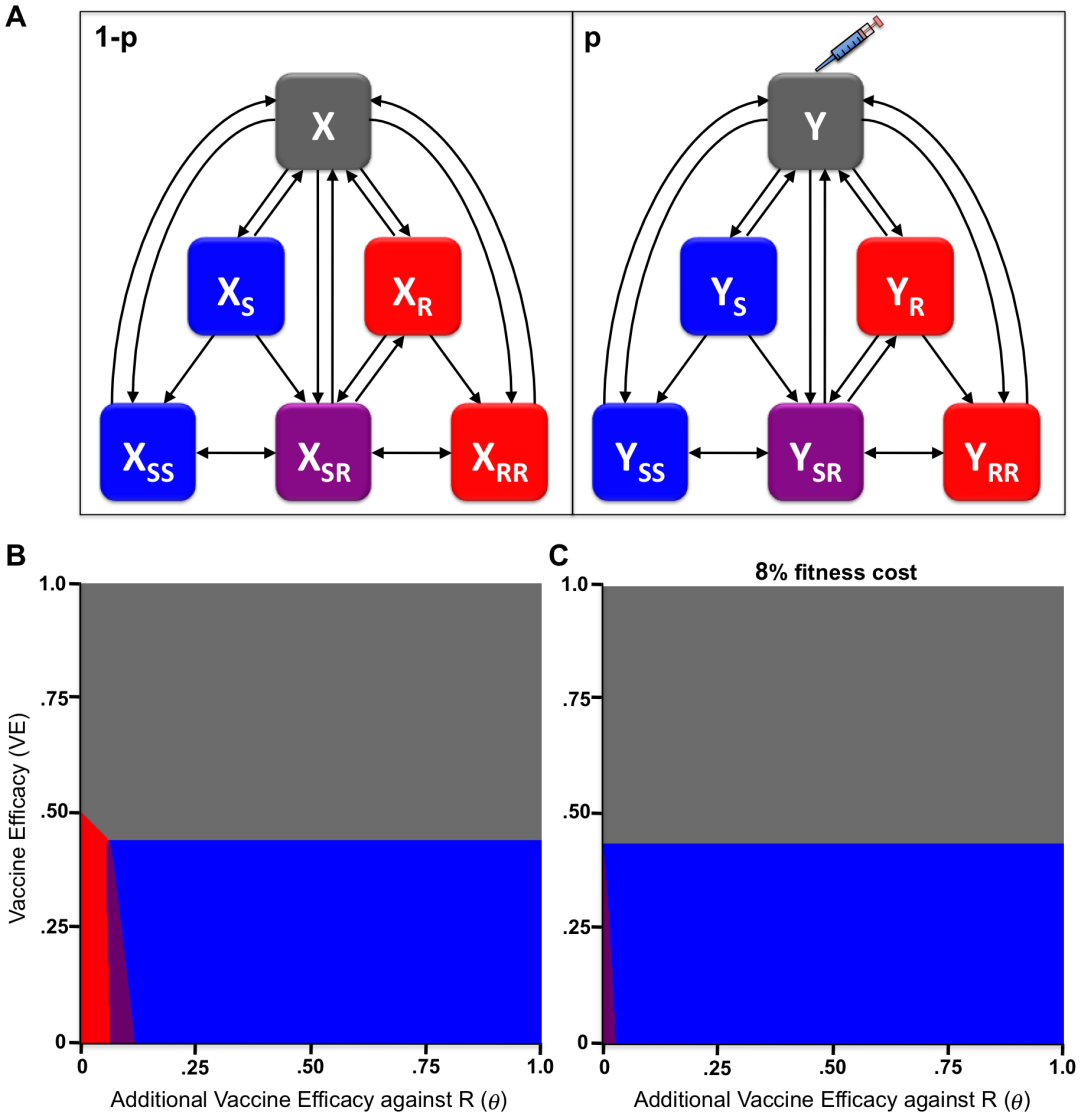
Modeling a vaccine with increased efficacy against drug-resistance determinants for an endemic colonizing pathogen (*S.* *pneumoniae*). **a**, SIS model with a proportion 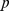 of the population vaccinated and initially susceptible (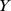) and 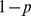 unvaccinated and initially susceptible (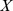), who can get infected with either the drug-sensitive strain (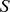 subscript), –resistant strain (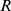 subscript), or both (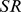 subscript) strains. Plots depict model state at equilibrium (all drug-resistant, all drug-sensitive, stable co-existence of both strains, or elimination of all strains) across a range of overall vaccine efficacy (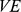) and additional vaccine efficacy against resistant strain (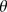), where vaccine coverage is 80%. Plots show situation with no fitness cost (**b**) and with 8% fitness cost (**c**). Color scheme throughout the paper is as follows: uninfected (gray), sensitive (blue), resistant (red), co-infected with both strains/coexistence of both strains (purple). This model corresponds to Model E of Ref [11].

To assess the impact of a resistance targeting vaccine on an endemic, colonizing pathogen in which resistant strains currently coexist with drug-sensitive strains, we considered the example of a pneumococcal vaccine that preferentially immunizes against penicillin-resistant variants, based on a structurally neutral co-colonization model of strain coexistence [11] (Figure 1A). We evaluated the model's equilibrium state across a range of values for overall vaccine efficacy (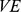) and the increase in vaccine efficacy against the resistant strain (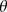). By varying 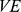 and 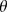, we identified conditions under which (i) drug-resistance reaches 100%, (ii) drug-sensitivity reaches 100%, (iii) co-existence of drug-resistant and -sensitive strains occurs, or (iv) both strains are eliminated. If there was no fitness cost to resistance, drug-resistance would reach 100% at baseline and between 7 and 13% additional resistant-strain-specific vaccine efficacy against the drug-resistant strain would be required to eliminate it (Figure 1B). However, a fitness cost of resistance (which has been shown to occur for penicillin resistance in pneumococci [36] and for many other resistance mechanisms in diverse microbes [37]) partially offsets the effect of high antibiotic usage, resulting in the coexistence of resistant and sensitive strains at baseline, as presently observed for penicillin resistance in *S. pneumoniae* [11]. Here, as additional vaccine efficacy against the drug-resistant strain (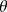) increases, drug resistance eventually disappears, outcompeted by the sensitive strain (Figure 1C). We find that for drug resistance to be eliminated, the vaccine needs only an additional 1–4% resistant strain-specific vaccine efficacy, given overall 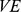 of up to 40% against all pneumococci.

**Figure 2:**
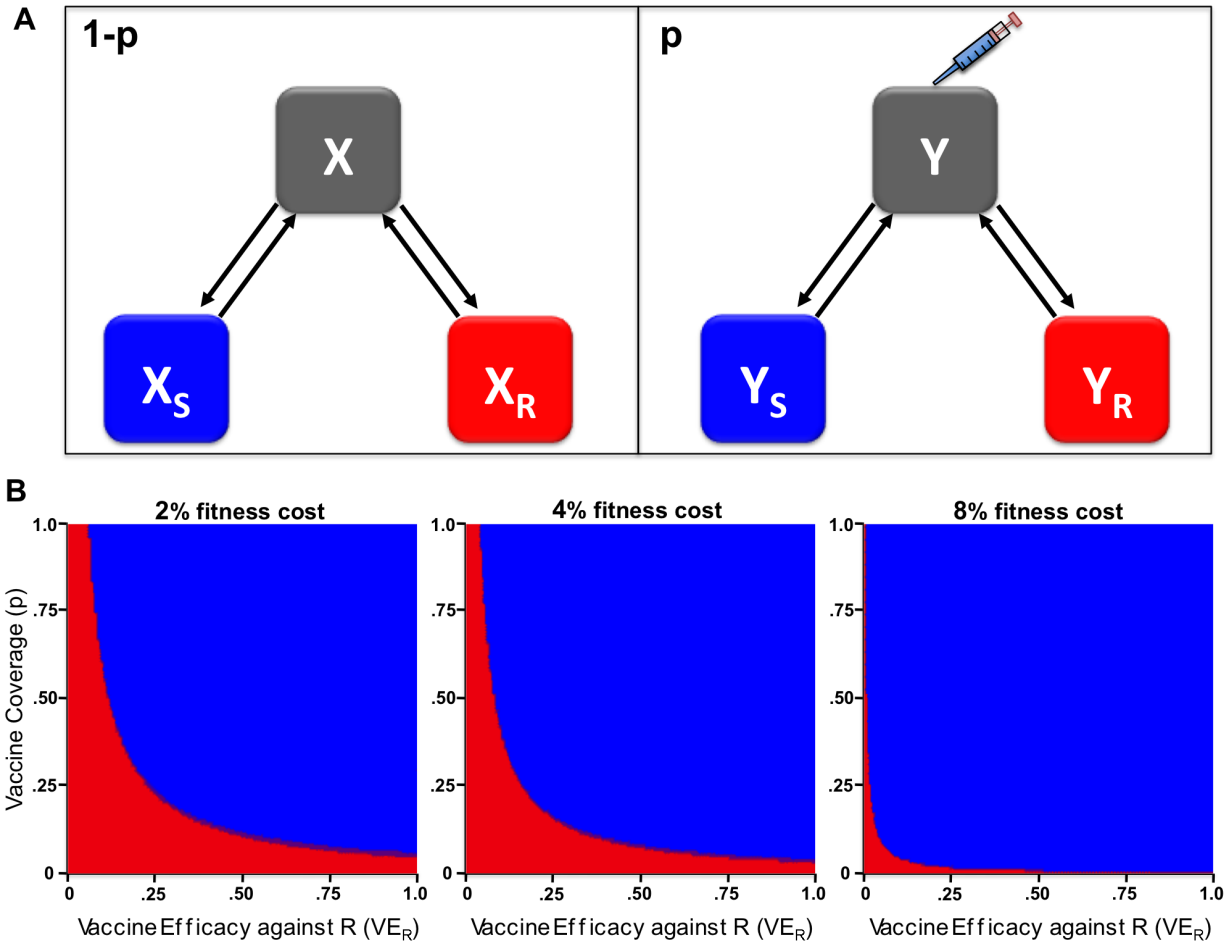
Modeling a vaccine against drug-resistance determinants for an endemic colonizing pathogen for which no vaccine currently exists (*S.* *aureus*). **a**, SIS model with a proportion 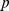 of the population as vaccinated susceptibles (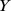) and 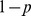 as unvaccinated susceptibles (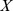), who can get colonized with either the drug-sensitive (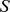 subscript), or –resistant (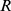 subscript) strain. **b**, Contour plot of equilibrium stability conditions as a function of vaccine coverage (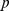) and specific vaccine efficacy against resistant strain (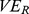), for 3 fitness costs. Stability conditions for the resistant-only and sensitive-only equilibrium were obtained analytically and were mutually exclusive. The stable equilibrium state is plotted by color as a function of fitness cost (different panels), vaccine efficacy against the resistant strain (x-axis) and vaccine coverage (y-axis).

Next, we considered a pathogen for which no vaccine currently exists, and for which the introduction of a drug-resistance-targeting vaccine could occur in the absence of a general acting vaccine for that pathogen. Community-associated (CA) *Staphylococcus aureus* is an endemic colonizing pathogen with high prevalence (carriage in 14 to 36% of healthy study cohorts) and rising rates of methicillin resistance (5% to 45% of carriers) [38], [39], [40], [41], [42]. Here we used a simpler single strain colonization model (Figure 2A) in which the vaccine exerts an effect against drug-resistant (methicillin-resistant *S. aureus*, MRSA) strains only, with no vaccine effect on drug-sensitive (methicillin-susceptible *S. aureus*, MSSA) strains. To test multiple vaccine mechanisms, we modeled either a reduced risk of acquisition (Figure 2B), or accelerated clearance rate upon getting colonized (Figure S1). We determined conditions for the stability of each equilibrium state and found that resistant strains are specifically eliminated when 
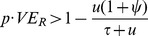
, where 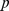 is vaccine coverage, 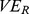 is MRSA-specific vaccine efficacy, 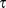 is the rate at which treatment clears colonization of MSSA, 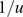 is the mean duration of colonization in untreated hosts, and 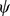 is the fitness cost of resistance. Thus drug-resistance is eliminated when the overall effect of the vaccine against MRSA (coverage×efficacy) is greater than the overall benefit of drug resistance, given as 1 minus the ratio of MRSA clearance rate to MSSA clearance rate. We assessed a range of vaccine efficacies (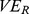) and coverage (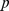) required to eliminate resistance and found that if the vaccine had high coverage (80%), only a marginal vaccine effect (0.6–7.5%) would be required to eliminate drug-resistance (Figure 2B). These modest MRSA-specific vaccine effects are in the same range as the 1–7% fitness costs that are believed to have led to a reduction of MRSA in Denmark following reduced antibiotic usage [34]. A variety of mechanisms lead to resistance in *S. aureus* (drug-inactivating enzymes, mutated PBP/target site and high expression of efflux pumps [20]), and depending on antigenicity and distribution in MRSA and MSSA clones, could serve as potential candidates for such a vaccine. Indeed, even if methicillin resistance itself could not be effectively targeted, there would be therapeutic benefit in maintaining the susceptibility of *S. aureus* to alternative drugs, as was initially the case with most CA-MRSA [43]. Alternatively or in addition, partially effective immunization could be achieved against factors associated with successful CA-MRSA clones, such as various staphylococcal toxins [44] which may even be genetically linked to methicillin resistance [45]; a caveat (see Discussion) to targeting linked factors rather than resistance determinants themselves is that these linkages might not persist once selection by the vaccine is in place, and the effect on resistance might be transient. High coverage rates could be achieved by combining this vaccine with a routinely given childhood vaccine.

**Figure 3:**
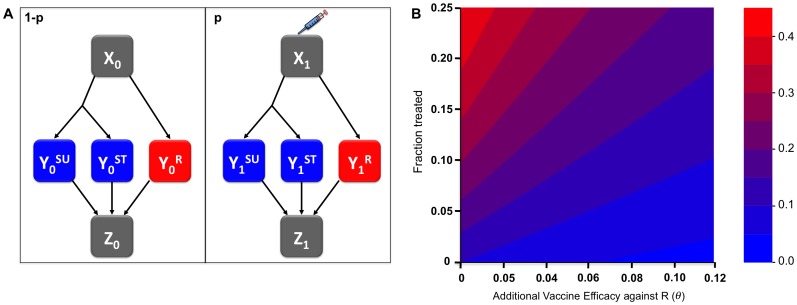
Modeling a vaccine with increased efficacy against drug-resistance determinants for an epidemic pathogen (seasonal influenza). **a**, SIR model with a proportion 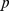 of the population as vaccinated susceptibles (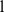 subscript) and 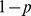 as unvaccinated susceptibles (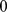 subscript), who can get infected with either the drug-sensitive (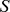 superscript) or –resistant (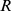 superscript) strains, get treated (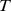 superscript) or not (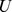 superscript) and are removed due to recovery or death (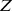). This is the model of Ref. [2], modified to include vaccination. **b**, Model evaluations for final cumulative proportion resistant among all infections over the course of one season, as a function of the additional vaccine efficacy 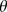 against resistant, compared to sensitive strains (x-axis) and the fraction of influenza infections treated (y-axis). Here, vaccine coverage is 40% and 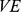 = 59%.

Last, we considered deployment of a killed influenza vaccine that includes a drug-resistant version of the neuraminidase (as a supplement to the hemagglutinin that forms the majority of antigenic material in current vaccines). We modeled a scenario (analogous to that at the end of the 2007–8 influenza season in some countries) in which a transmissible strain resistant to a neuraminidase inhibitor has been identified but has not yet reached fixation in a particular population (Figure 3A). A vaccine incorporating this resistant neuraminidase, we assumed, offers marginally greater protection against resistant infection than against infection with a drug-sensitive strain. We assumed that 10% of the cases that seeded the epidemic at the start of the season were resistant (due to importation) and as a worst-case scenario that resistance had no intrinsic fitness cost. We found that a vaccine of 7% increased efficacy against the resistant strain (66% efficacy vs. 59% against the sensitive strain) could counteract the effect of treating approximately 10% of the population with oseltamivir (Figure 3B). In 2007, the spread of oseltamivir resistance may have been mainly due to the appearance of a fitness benefit associated with the resistant strain, even in the absence of treatment [46], rather than by antiviral use. A resistance vaccine could also slow the spread of resistant virus in such a scenario (Figure S3).

## Discussion

Vaccines targeting drug-resistant determinants can have a substantial impact at the population level by reducing the competitive advantage held by these microbes due to the selective effect of drug pressure. These simplified models abstract away important features of transmission in each pathogen, including strain-specific immunity, host heterogeneity, and transmission between countries that might have different vaccination policies; thus precise predictions of the quantitative effects of such vaccines would depend on more detailed models of both pathogen transmission and vaccine effect. Nonetheless, with varying structures and assumptions, these models show that with high coverage, a vaccine with extremely modest effects (well below those typically sought for vaccines) could retard the spread of drug resistance in a range of pathogens.

The benefit of a resistance vaccine depends on specific increased efficacy against the resistant type. Recent work on Th17-based immunity to pneumococci shows that antigen-specific immunity elicited in the setting of dual carriage of antigen-bearing and antigen-lacking pneumococci leads to near-equal clearance of both strains, because the activation (via T cells) but not the effector cells (neutrophils) are antigen-specific [47]. Such a vaccine would have lower effectiveness in suppressing resistance because its additional effect against the resistant strain would be realized in hosts colonized with only the sensitive or resistant strain, but not in hosts co-colonized with both. Vaccines based on antibodies, CD8+ T cells, or other mechanisms that target effectors to antigen-positive cells would not have this limitation.

The amount of resistance-specific efficacy required to eliminate drug-resistance depends on a range of parameters relating to the biology of the pathogen (duration of colonization, fitness cost of drug-resistance), the degree to which interventions are used (treatment rate, vaccine coverage), and the overall efficacy of vaccination against all strains. For each of the organisms we modeled, modest vaccine efficacies were required to eliminate resistance. Despite differences in parameter values (Table 1) and overall vaccine efficacy (ranging between 0–59%), we predict that between 1–13% resistance-specific efficacy is required to eliminate resistance in each system, given an 80% vaccination coverage rate. As treatment rate and duration of infection are not well understood for *S. aureus*, we tested a range of parameter values to encompass the range reported in the literature (Table S1 in File S1). Higher resistance-specific vaccine efficacies were required to eliminate resistance when treatment rates or durations of colonization increased, or fitness costs were reduced (Figure S2 and Table S1 in File S1). However, across the entire range of parameters tested, the resistant-specific efficacy required to eliminate drug-resistance never surpassed 30%. While additional research into these parameter values could help the model's accuracy, the overall result still suggests a modestly effective vaccine is capable of eliminating drug-resistance if given at high coverage.

Comparing our results with those from prior modeling studies on using vaccination to target drug-resistance, we are the first to show that targeting of drug-resistance determinants can lead to sustained reduction in drug-resistance population-wide. Vaccination with resistance determinants themselves would slow, if not prevent, the process by which resistance determinants spread in strains where the vaccine-targeted antigens are absent, as modeled in Temime et al [27] and happened within several years of pneumococcal conjugate vaccination in the US [14], [29]. We predict that 1–13% resistant-specific vaccine efficacy is required for eliminating drug resistance, which is substantially lower than that shown in Tekle et al, in which 56–83% resistance-specific vaccine efficacy was required to eliminate drug-resistance in hospital settings [28]. This difference could be explained due to our focus on vaccination in the community. Vaccination in the community is a more attractive option for reducing drug-resistance population-wide than vaccination prior to hospital admission because (1) vaccines against colonizing organisms generally prevent colonization better than they induce clearance of colonization once established [48]; and (2) durations of hospital stays, which average about 5 days in the US [49] are shorter than the typical time required for vaccination to elicit protective immunity (weeks).

One caveat of our approach is that drug-resistance in a single organism can arise through a variety of mechanisms and thus drug-resistance conferring proteins, meaning that if vaccines target only one drug-resistance conferring protein, microbes expressing alternate proteins that confer resistance could spread in the population. In several of the organisms we explore, multiple mechanisms and proteins are involved in conferring drug-resistance. For this intervention to succeed, drug-resistance vaccines should only be used for organisms in which vaccines can be generated against epitopes covering the range of resistance-conferring proteins for that organism. Alternatively, they could be designed for organisms with only one known resistance-conferring mechanism.

Current methods for combating drug-resistance include the development of new drugs to kill drug-resistant microbes, and new drug combinations to prevent *de novo* evolution of drug-resistance. Microbes have been shown to acquire drug-resistance at alarming rates, which requires the continuous development of new antimicrobials in order to keep up with this arms race. While high throughput drug discovery programs are useful in this process, getting new drugs approved is a long and expensive process. Further, there are no guarantees that we can keep up, as evidenced by the virtually untreatable forms of extensively drug resistant tuberculosis (XDR-TB) that have cropped up in recent years. Thus, the use of ecological-focused interventions that attempt to steer microbial populations toward drug-sensitive infections rather than drug-resistant ones are a favorable alternative. Campaigns to reduce antibiotic usage and thus reduce selective pressure for drug-resistance have succeeded in the reduction of drug-resistance in some settings [34]. Likewise we show that the use of a vaccine targeting resistant strains can combat the selective pressure for drug-resistance.

Mechanistically, a key concept is that the presence of drug-sensitive, competing strains enhances the herd immunity effects of the vaccine against the resistant strain [50] and allows large population-level effects despite low efficacy. Such competition has been documented for strains of *S. aureus* [51], [52] and *S. pneumoniae* (for which the best evidence of competition is the existence of serotype replacement following serotype-specific vaccines [53]); for influenza, competition results from the immunity to influenza reinfection that occurs within a season [54]. In the presence of competition between sensitive and resistant strains, the resistance vaccine alone does not have to bring the reproductive number below 1 to lead to elimination of the resistant strain, as for classical vaccines against monomorphic pathogens [55] but only below that of its competitor, the sensitive strain.

Vaccine designs for drug-resistant pathogens should take into account the notion that even a weakly effective vaccine may create enough of a competitive disadvantage for drug-resistant strains to facilitate the sensitive strains in outcompeting them population-wide, despite substantial antimicrobial use. While vaccine design and approval processes may typically reject the notion of weakly effective vaccines, we show here that their use as an ecological intervention against drug-resistance in the population can be profound. Further, the deployment of such a vaccine at high coverage rates is not unimaginable, as it could be combined into routine childhood vaccinations.

## Supporting Information

Figure S1In addition to a vaccine that reduces susceptibility to acquisition (shown in Main Text Figure 2B), for comparison we considered vaccine that works via accelerated clearance (as possibly expected for T-cell-mediated immunity) of *S. aureus*. Contour plot of equilibrium stability conditions as a function of vaccine coverage (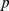) and specific vaccine efficacy against resistant strain (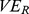), for 3 fitness costs. Stability conditions for the resistant-only and sensitive-only equilibrium were obtained analytically and were mutually exclusive. The stable equilibrium state is plotted by color as a function of fitness cost (different panels), vaccine efficacy against the resistant strain (x-axis) and vaccine coverage (y-axis).(TIF)

Figure S2 In order to test a broader range of parameters as some of parameter values (particularly treatment rate and duration of infection) are not well understood for *S. aureus*, we varied the treatment rate (from 10–100% of MSSA-active antibiotic prescriptions per person per week) and the clearance rate (from 175–700 days, consistent with range of durations reported in studies of drug-sensitive or resistant *S. aureus* carriage in the nose and throat [56], [57]). Here, we used the reduced susceptibility vaccine and a range of fitness costs, 2% (a), 4% (b), and 8% (c).
(TIF)

Figure S3Ability of a resistance vaccine against influenza to counteract the spread of a resistant strain due to an intrinsic fitness advantage, not due to antimicrobial use. Here the total proportion resistant over a season is plotted as a function of the additional vaccine efficacy against the resistant strain 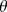 and the intrinsic fitness advantage of the resistant strain, estimated at about 2% for the influenza A/H1N1 strain carrying the H275Y neuraminidase mutation in 2006–9 [46].(TIF)

File S1 Supporting Methods.
(DOCX)
