# Supplementary figures and images for: Targeting Imperfect Vaccines against Drug-Resistance Determinants: A Strategy for Countering the Rise of Drug Resistance
Source: PLoS One. 2013 Jul 25;8(7):e68940. doi: 10.1371/journal.pone.0068940 (PMC3723804; doi:10.1371/journal.pone.0068940)

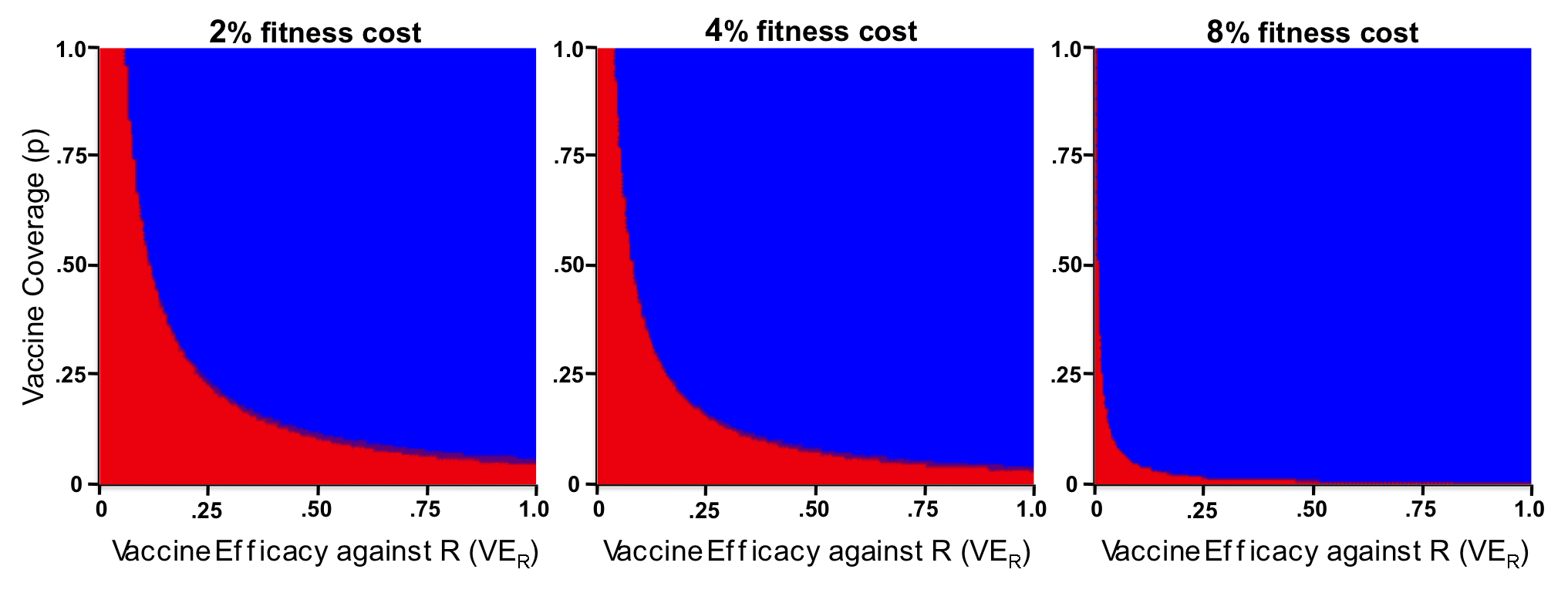

Supplement: Figure S1 — In addition to a vaccine that reduces susceptibility to acquisition (shown in Main Text Figure 2B), for comparison we considered vaccine that works via accelerated clearance (as possibly expected for T-cell-mediated immunity) of S. aureus. Contour plot of equilibrium stability conditions as a function of vaccine coverage () and specific vaccine efficacy against resistant strain (), for 3 fitness costs. Stability conditions for the resistant-only and sensitive-only equilibrium were obtained analytically and were mutually exclusive. The stable equilibrium state is plotted by color as a function of fitness cost (different panels), vaccine efficacy against the resistant strain (x-axis) and vaccine coverage (y-axis). (TIF) [file pone.0068940.s001.tif]

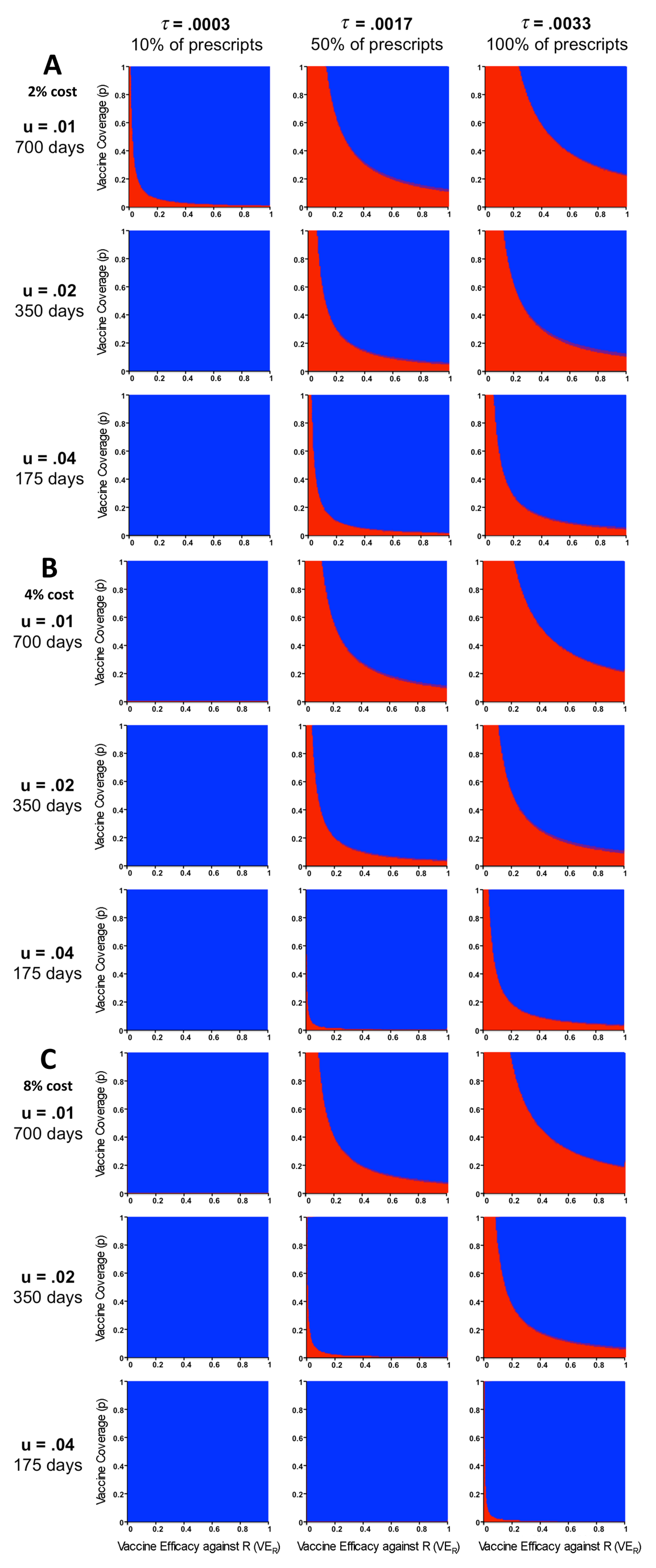

Supplement: Figure S2 — In order to test a broader range of parameters as some of parameter values (particularly treatment rate and duration of infection) are not well understood for S. aureus, we varied the treatment rate (from 10–100% of MSSA-active antibiotic prescriptions per person per week) and the clearance rate (from 175–700 days, consistent with range of durations reported in studies of drug-sensitive or resistant S. aureus carriage in the nose and throat [56], [57]). Here, we used the reduced susceptibility vaccine and a range of fitness costs, 2% (a), 4% (b), and 8% (c). (TIF) [file pone.0068940.s002.tif]

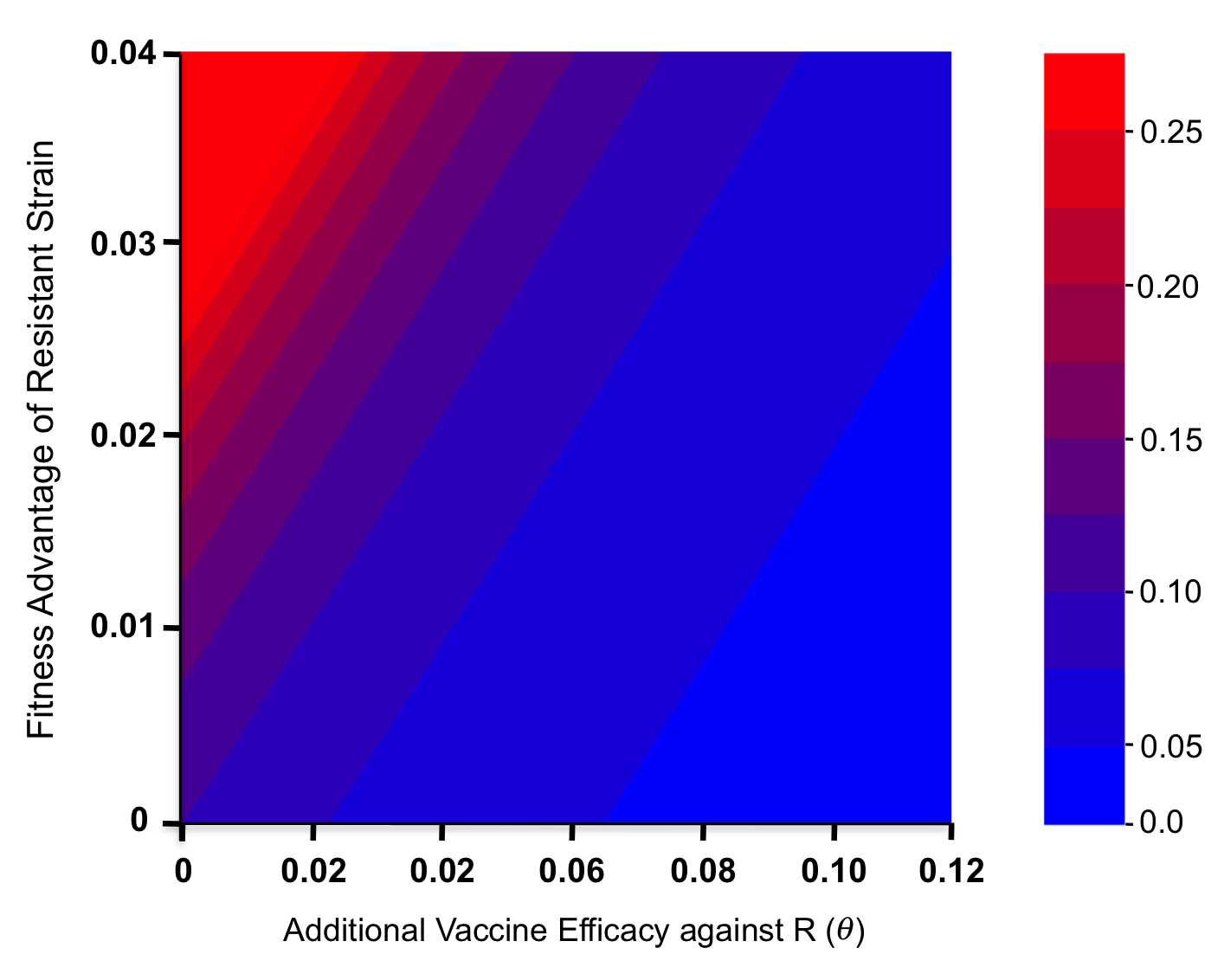

Supplement: Figure S3 — Ability of a resistance vaccine against influenza to counteract the spread of a resistant strain due to an intrinsic fitness advantage, not due to antimicrobial use. Here the total proportion resistant over a season is plotted as a function of the additional vaccine efficacy against the resistant strain and the intrinsic fitness advantage of the resistant strain, estimated at about 2% for the influenza A/H1N1 strain carrying the H275Y neuraminidase mutation in 2006–9 [46]. (TIF) [file pone.0068940.s003.tif]
