# Supplementary material for: Targeting Imperfect Vaccines against Drug-Resistance Determinants: A Strategy for Countering the Rise of Drug Resistance
Source: PLoS One. 2013 Jul 25;8(7):e68940. doi: 10.1371/journal.pone.0068940 (PMC3723804; doi:10.1371/journal.pone.0068940)
Supplement: File S1 — Supporting Methods. (DOCX) [file pone.0068940.s004.docx]

**Supporting Methods**

***I. S. pneumoniae***

**A. Model Structure.** The model employed is based on a previously published structurally neutral model of competitive dynamics between drug-susceptible (
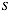
) and drug-resistant (
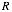
) *S. pneumoniae*[11]. In this model, hosts can be colonized by one or two strains simultaneously, allowing them to be in one of 6 different states (
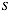
,
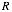
,
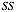
,
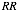
,
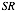
 or none). We extend the earlier model to include vaccination by having 2 types of individuals in the population, those that are vaccinated (a proportion
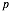
) and those that are not (
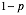
), which we fix in the model. While an individual’s vaccination status cannot change (no entry/exit from vaccinated classes), their colonization state is affected by the presence of
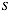
 and
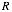
 strains in the population (force of infection takes into account all infecteds in the population). We model the vaccine efficacy against the drug-sensitive strain as
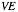
, and model the vaccine efficacy against the resistant strain as
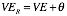
, where
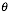
 is the additional resistant-strain-specific efficacy. This system of equations describes the dynamics of the model. Parameters are listed in Table 1, with the exception of
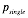
,
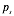
,
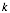
 and
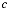
, which have previously been described[11], and were all set to 0.5 in this model. Categories of unvaccinated individuals are represented by an
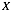
, while categories of vaccinated individuals are represented with a
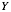
. Subscripts are as follows: single drug-sensitive (
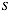
), single drug-resistant (
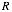
), dual drug- susceptible (
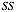
), dual drug-resistant (
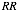
) and dual mixed with drug-sensitive and drug-resistant (
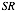
).

The equations are:


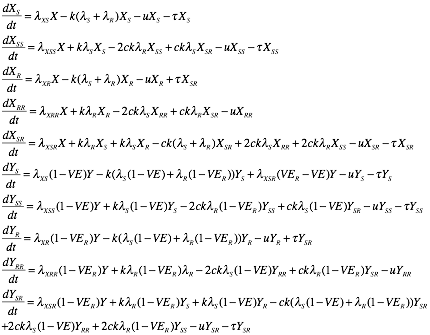


where:


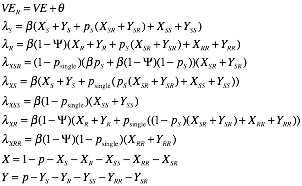


**B. Equilibrium Plots.** Equilibrium values were plotted using Mathematica version 8.0. The models were run for 2000 weeks under each parameter combination, and the total number of
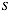
-colonized and
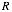
-colonized individuals were tabulated for each parameter value. For each (
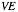
,
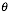
) point at increments of 0.0025, we determined whether any individuals were present at equilibrium in an
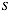
 and/or
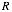
 category using a cutoff of 0.001 (i.e. if less than 0.1% of the population was
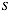
- or
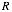
-colonized, that category was designated absent). If all individuals were
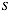
 but none
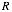
, we designated the block on the array plot as
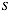
-only equilibrium (blue), if all
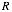
 but none
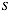
, we designated
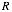
-only (red), if both, co-existence (purple) and if neither, the infection did not take off (gray). The behavior of this model has been previously analyzed[11].

***II. S. aureus***

**A. Model Structure.** We used a SIS model in which individuals can be colonized by either a drug-sensitive or a resistant strain (no co-colonization). We considered a vaccine targeted only against drug-resistant strains (no vaccine effect on sensitive strains). To test multiple vaccine mechanisms, we modeled both a vaccine that reduces the risk of acquisition for a vaccinated individual (as might be expected for a vaccine that elicits antibody) or one that hastens clearance for vaccinated individuals (as might be expected for a vaccine that elicits T cells[63]). We again model vaccination by having 2 types of individuals in the population, those that are vaccinated (
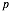
), and those that are not (
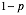
), assuming a closed host population and no changes to vaccine status over time. Force of infection takes into account all infecteds in the population (both vaccinated and not). This system of equations describes the dynamics of the model. Parameters are listed in Table 1.

The equations for mechanism 1 (reduced transmission) are:


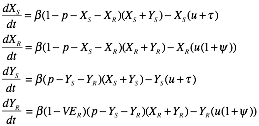


The equations for mechanism 2 (rapid clearance) are:


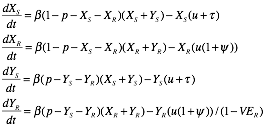


**B. Treatment Rate Calculations.** We estimated treatment rate of drug-sensitive *S. aureus* using antibiotic prescribing and drug-resistance data for *S. aureus*. Using antibiotic prescribing data from 2005-2006 from Ref. [33], we calculated the weighted average number of antibiotics in all visits per person per year, weighted based on the 2010 U.S. census data of age group size breakdown[64]. We then used the breakdown of prescriptions by drug class prescribed during acute respiratory tract infection visits[33] to adjust this number to the fraction of total antibiotic prescriptions per year per person that we estimate will be active against methicillin-sensitive but not methicillin-resistant community-associated *S. aureus*. Based on drug-susceptibility data in *S. aureus*[65] we considered the MSSA-specific fraction of antibiotic prescriptions to contain amoxicillin/clavulanic acid, cephalosporins, and quinolones, and not to include macrolides and sulfonamides. We then took the weighted average number of MSSA-specific antibiotic prescriptions per person per week and took 50% of this number as our estimate of treatment rate since treatment is not always curative. In the supplement, we explore higher and lower estimates (100% and 10% of prescriptions, respectively), see Figure S2.

**C. Equilibrium Plots.** Mathematica version 8 was used to explore equilibrium dynamics of the systems of equations for both vaccine mechanisms (reduced transmission and rapid clearance).  We first determined the equilibrium states by setting all equations to zero and solving for
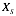
,
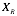
,
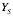
, and
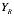
, which resulted in four nonnegative solutions, verified arithmetically using a set of parameter values. These four equilibrium states were (a) transmission is eliminated (all state variables = 0), (b) the drug-sensitive strain alone is present (
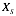
 and
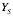
 are positive, while
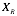
 and
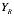
 are zero), (c) the drug-resistant strain alone is present (the opposite), and (d) both drug-sensitive and drug-resistant strains coexist (all state variables are positive). The stability of these equilibria was then evaluated by determining conditions that implied the negativity of all eigenvalues of the Jacobian Matrix at a given equilibrium.   For the reduced transmission vaccine shown in Figure 2 and the rapid clearance vaccine shown in Figure S1, the stability conditions for the equilibrium in which infection does not take off at all were:
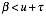
 and
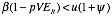
. These expressions correspond to the conditions that the basic reproduction numbers for both sensitive and resistant strains are less than one. The stability condition for the drug-sensitive equilibrium for both models was as follows:
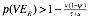
 (which is discussed in main text, and is also the condition necessary to selectively eliminate drug-resistance). For the other equilibria, eigenvalues were not calculated due to complexity of the system. However, because one of the side blocks of the 4x4 Jacobian matrix solved for the drug-resistance equilibrium was a zero matrix, we calculated traces and determinants of the two diagonal blocks to determine stability conditions (conditions under which the traces were negative and determinants were positive). We then plotted the eigenvalue stability conditions from the first two equilibria and the trace/determinant conditions from the diagonal blocks of the third equilibrium as functions of
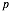
 and
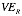
_,_ with other parameters held constant at the values listed in Table 1. Using a contour plot, we plotted these expressions to explore conditions under which the various equilibria are stable. If conditions were met for stability of the no-colonization equilibrium, we shaded gray (this was not observed in the current sets of parameters plotted), if S-only (blue), if R-only (red), and if none of the above conditions were met, we designated the co-existence equilibrium (purple).

***III. Influenza***

**A. Model Structure.** To study the role of resistance vaccines in influenza, we used a model previously published for antiviral resistance[2]. In a rapidly-spreading, immunizing infection such as influenza, an epidemiologically meaningful measure of the extent of resistance is the cumulative proportion of incident cases throughout the epidemic that are resistant[2].

We considered the situation in which resistant strains were already present as 10% of incident cases at the start of the epidemic, in the absence of a fitness cost to resistance, and assessed the effect of vaccinating 40% of individuals with a vaccine that is
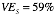
 protective against infection. Here, the S subscript refers to vaccine efficacy against susceptibility to infection[35] with the drug-sensitive strain,
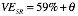
 protection against the resistant strain. We focus on seasonal influenza because for pandemic influenza, it is unlikely that one would have sufficient advance notice of a novel minority resistant strain to modify the vaccine accordingly; thus we assume that 30% of the population (all ages) is immune at the start of the season. The age-structured model (parameterized as in Ref. [2]) was used.

The equations are:


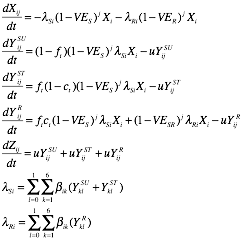


Here, the subscript *i* refers to age groups, and *j* to vaccination status (0=unvaccinated, 1=vaccinated).

In Main Text Figure 3, we show the effect of a resistance vaccine in counteracting selection by antiviral use. In Figure S3, we consider the alternative situation in which no antivirals are used, but the resistant strain has a 2% intrinsic fitness advantage (negative 2% fitness cost) [46].

**Additional References**

63. Miller LS, Cho JS (2011) Immunity against Staphylococcus aureus cutaneous infections. Nat Rev Immunol 11: 505-518.

64. US Census Bureau (2011) Data from Age and Sex Composition: 2010. 2010 Census Briefs. C2010BR-03.

65. Deleo FR, Otto M, Kreiswirth BN, Chambers HF (2010) Community-associated meticillin-resistant Staphylococcus aureus. Lancet 375: 1557-1568.

**Supporting Table**

**Table S1.** Vaccine efficacy against resistant strain (
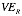
) required to eliminate drug resistant *S. aureus* across a range of fitness costs, durations and treatment rates, given 80% vaccine coverage.

|  | 2% fitness cost | | | 4% fitness cost | | | 8% fitness cost | | |
| --- | --- | --- | --- | --- | --- | --- | --- | --- | --- |
| Clearance Rate,  Duration | τ = .0003,  10% of Rx | τ = .0017,  50% of Rx | τ = .0033,  100% of Rx | τ = .0003,  10% of Rx | τ = .0017,  50% of Rx | τ = .0033,  100% of Rx | τ = .0003,  10% of Rx | τ = .0017,  50% of Rx | τ = .0033,  100% of Rx |
| *u* = .01,  700 days | 1.6% | 16.0% | 29.1% | - | 13.9% | 27.3% | - | 9.6% | 23.5% |
| *u* = .02,  350 days | - | 7.5% | 15.6% | - | 5.2% | 13.4% | - | 0.6% | 9.1% |
| u = .04,  175 days | - | 2.7% | 7.2% | - | 0.3% | 4.9% | - | - | 0.3% |
